# BioVerbNet: a large semantic-syntactic classification of verbs in biomedicine

**DOI:** 10.1186/s13326-021-00247-z

**Published:** 2021-07-15

**Authors:** Olga Majewska, Charlotte Collins, Simon Baker, Jari Björne, Susan Windisch Brown, Anna Korhonen, Martha Palmer

**Affiliations:** 1 Language Technology Laboratory, MMLL, University of Cambridge, 9 West Road, Cambridge, CB39DB UK; 2 Department of Future Technologies, University of Turku, Vesilinnantie 5, Turku, 20500 Finland; 3 Department of Linguistics, University of Colorado Boulder, 295 UCB, Boulder, 80309-0295 Colorado USA

**Keywords:** Verb lexicon, VerbNet, Text classification

## Abstract

**Background:**

Recent advances in representation learning have enabled large strides in natural language understanding; However, verbal reasoning remains a challenge for state-of-the-art systems. External sources of structured, expert-curated verb-related knowledge have been shown to boost model performance in different Natural Language Processing (NLP) tasks where accurate handling of verb meaning and behaviour is critical. The costliness and time required for manual lexicon construction has been a major obstacle to porting the benefits of such resources to NLP in specialised domains, such as biomedicine. To address this issue, we combine a neural classification method with expert annotation to create BioVerbNet. This new resource comprises 693 verbs assigned to 22 top-level and 117 fine-grained semantic-syntactic verb classes. We make this resource available complete with semantic roles and VerbNet-style syntactic frames.

**Results:**

We demonstrate the utility of the new resource in boosting model performance in document- and sentence-level classification in biomedicine. We apply an established retrofitting method to harness the verb class membership knowledge from BioVerbNet and transform a pretrained word embedding space by pulling together verbs belonging to the same semantic-syntactic class. The BioVerbNet knowledge-aware embeddings surpass the non-specialised baseline by a significant margin on both tasks.

**Conclusion:**

This work introduces the first large, annotated semantic-syntactic classification of biomedical verbs, providing a detailed account of the annotation process, the key differences in verb behaviour between the general and biomedical domain, and the design choices made to accurately capture the meaning and properties of verbs used in biomedical texts. The demonstrated benefits of leveraging BioVerbNet in text classification suggest the resource could help systems better tackle challenging NLP tasks in biomedicine.

## Background

The demand for automatic systems capable of processing and mining the rapidly expanding body of biomedical literature is constantly growing and NLP technologies can play a key role in facilitating the dissemination and consolidation of knowledge recorded in scientific papers, patient reports, or clinical notes. The domain-specific properties of biomedical texts require specialised systems sensitive to the well-defined semantics and syntactic behaviour of the terms used in the scientific literature. This is why high-quality, rich computational lexicons comprising information about the meaning and combinatorial properties of words in biomedical texts can significantly boost the performance of NLP systems in problems ranging from information retrieval, relation and event extraction, or entailment detection. Similarly to the general language domain, lexicographic efforts in biomedicine have primarily focused on nouns (e.g., UMLS Metathesaurus [1]), while the demand for rich, large-coverage verb-specific biomedical resources has not yet been satisfied [2–6].

A number of works in general domain NLP have illustrated the benefits offered by databases of structured verb-related knowledge. One such resource is VerbNet [7], a broad-coverage, hierarchical classification of English verbs providing detailed documentation of verbs’ semantic and syntactic properties. It has been successfully employed to boost NLP tasks such as word sense disambiguation [8], semantic role labelling [9], information extraction [10], or text mining [11, 12]. While the utility of VerbNet in the general domain has been widely recognised, the lexicographic effort involved in its construction poses a challenge to transferring its benefits to other specialised domains.

In this work, we address the demand for a biomedical verb resource and alleviate the issue of slow manual dataset construction by combining a data-driven automatic classification approach with post-hoc expert verification and annotation to create the first BioVerbNet. We take the output of a highly accurate neural classification approach of Chiu et al. [13] as a starting point and subsequently manually validate the resultant classes based on VerbNet class criteria of semantic-syntactic coherence of member verbs. The 22 top-level and 117 fine-grained verb classes produced in the process are then annotated with semantic roles of the verbs’ arguments and syntactic frames in which class members participate, thus yielding a rich semantic-syntactic lexicon of biomedical verbs.

The creation of BioVerbNet involved the following key stages. First, the 1149 verbs assigned to 50 classes by the system of Chiu et al. were reviewed by a domain expert and a linguist, to identify noisy candidates to be eliminated from the classification. Next, each class was individually validated by verifying each individual candidate member’s consistency with the rest of the class in terms of closeness of meaning and shared structural properties, based on the most frequent dependency contexts extracted from the PubMed corpus [14] by Chiu et al. In the process, the experts decided whether misclassified candidates should be (a) reassigned to another existing class, (b) assigned to a new class, or (c) discarded from the classification. We examined the domain specificity of our classification by comparing BioVerbNet to VerbNet, which revealed a very limited coverage of biomedical verbs in VerbNet and important discrepancies in the dominant senses represented by the verbs shared by both resources. Next, for each class a set of representative syntactic contexts was selected, each subsequently annotated with syntactic descriptions, capturing the possible surface realisations of the member verbs’ arguments, and their semantic roles. In order to better capture the characteristic properties of the entities acting as Agents in biomedical scenarios (e.g., cells, chemical reactions, biological processes), we introduced a new biomedicine-specific role of *Bio-Agent*, distinct from the canonical agentive arguments (e.g., human actors) in the general language domain.

We demonstrate the utility of the newly created verb lexicon to support neural approaches to two biomedical text classification tasks. We derive verb class knowledge in the form of pairwise constraints extracted from the BioVerbNet classification, which we employ to retrofit the vector space of pretrained word embeddings to better reflect the shared semantic-syntactic properties represented by each verb class by pulling co-members closer together. We input the BioVerbNet-specialised embeddings into a convolutional neural network model and evaluate it on document- and sentence-level classification tasks using two established biomedical datasets, the Hallmarks of Cancer [15] and the Exposure taxonomy [16]. The promising results achieved by the model boosted with BioVerbNet class information holds promise for future applications of the resource in downstream tasks in biomedicine.

## Related work

### Computational verb resources

English-language general domain NLP has a number of large-scale expert-built resources at its disposal from which to derive rich information about verb behaviour. These include, among others, the large semantic network WordNet [17], FrameNet [18], which organises concepts in the so-called semantic frames, describing different types of events, relations, entities, and their participants, and PropBank [19], which includes information about semantic propositions and predicate-argument structure of verbal predicates. A lexicon focused exclusively on verbs is VerbNet, which extends Levin’s [20] taxonomy of English verbs and groups them into classes based on shared semantic-syntactic properties. Each such class is accompanied by a set of frames, including a syntactic description and a semantic representation, as well as thematic roles and selectional restrictions on the verbs’ arguments. VerbNet classes capture useful generalisations about verb behaviour and can boost NLP systems’ predictive capacity on unseen vocabulary by providing a means of extrapolating from individual word types to classes. For instance, by linking an unseen verb *quell* to its class SUBJUGATE, a system can refine its meaning representation to align more closely with other, seen class members with higher occurrence rates in corpora (e.g., *suppress, dampen*). VerbNet has been used as a source of syntactic and semantic features supporting a range of NLP applications, including machine translation [21], semantic parsing [22], word sense disambiguation [8, 23], information extraction [10] and text mining [11, 12]. While VerbNet offers vast coverage (it currently includes 9344 verbs organised in 329 main classes), its utility cannot be directly extended to specialised domains, such as biomedicine, where verbs occur in domain-specific senses and distinct contexts, different from their patterns of behaviour in general English. This is why creation of resources tailored to the characteristics of biomedical texts and terminology is essential.

### Biomedical lexicons

A large biomedical lexical resource available is the UMLS Metathesaurus, the most-extensive thesaurus in this domain, which classifies concepts pertaining to biomedicine by semantic type and stores information about the relationships among them. While the resource has been used to support biomedical data mining and information retrieval, its focus is on nouns. The currently available verb-specific lexicons have much smaller coverage and are usually limited to narrow sub-domains. For instance, the manually-created UMLS SPECIALIST lexicon is focused on medical and health-related terminology, whereas the BioLexicon, which provides syntactic and semantic frame information for biomedical verbs, is extracted from the Escherichia Coli (E. Coli) corpora, which restricts its utility to this particular subdomain.

### Representation learning and text classification in BioNLP

With deep learning, representation learning has become a standard technique in natural language processing and also in biomedical natural language processing. The utilization of representation learning methods in BioNLP has followed the introduction of such methods in general NLP, and has usually been accompanied by efforts to adjust and specialize these methods for a biomedical vocabulary. In the development of BioNLP research during the past decade, the introduction of Word2Vec [24] has prepared the way for wider use of neural concept representations [25].

The most common use case for word vectors has been as input embeddings for deep learning neural networks, but word vectors have also been used directly for analysing concepts such as semantic similarity and relatedness [26]. Word vectors trained on general domain texts such as news articles may not always have captured the specific semantics of biomedical concepts, so the methods of Word2Vec, GloVe [27] and FastText [28] have been adapted for the generation of specialized vector space representations usually based on the PubMed collection of millions of biomedical research articles [29–31]. In the BioWordVec project [32] further information from MeSH (Medical Subject Headings) is used to augment PubMed text resources. Wang et al. [33] have shown that training embeddings specifically on biomedical text can produce more relevant vector space representations.

The introduction of generalized language models like ELMo and BERT [34, 35] with their integrated embedding vocabularies introduced a new, more unified approach for utilization of representation learning in language models. As with the word vector models, ELMo and BERT were also rapidly adapted for the specifics of biomedical language [36, 37].

With the advent of deep learning, representation learning has become a common technique in many text mining tasks such as classification. Neural networks based on convolutional and recurrent (especially LSTM) approaches have achieved significantly improved results on many biomedical text mining tasks [38–40].

Transformer models have resulted in even larger performance gains [25]. The BioBERT model itself demonstrated state of the art performance on named entity recognition, relation extraction and question answering. Since its publication this model has been applied to tasks such as drug–drug interaction extraction, classification of social media health discussions and analysis of scientific articles related to COVID-19 [41–43].

## Construction and content

### Dataset design

**Fig. 1:**
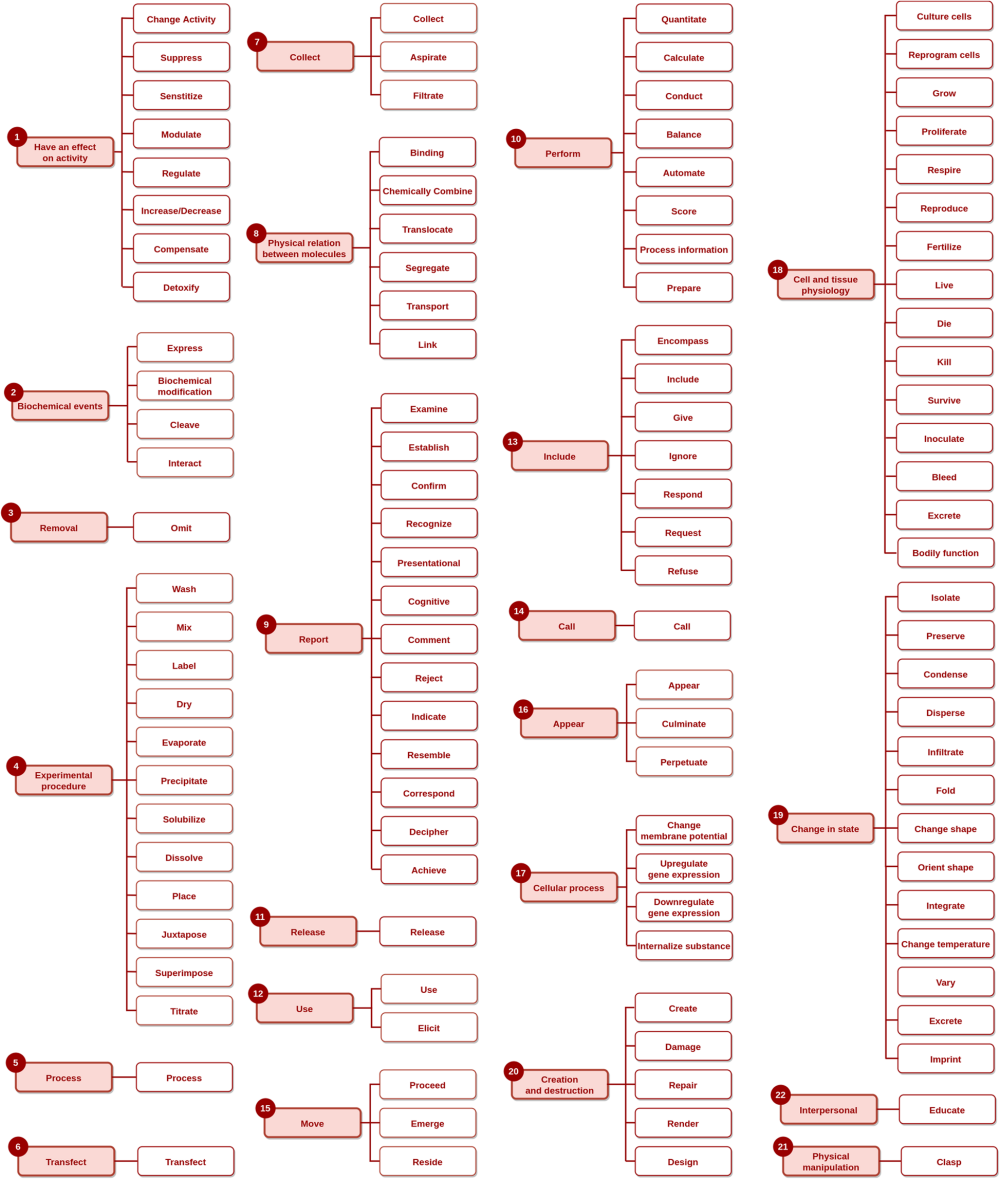
A visual representation of the BioVerbNet semantic classes. The shaded boxes represent the top-level classes, while the unshaded boxes represent the subclasses

The starting point for the construction of BioVerbNet is the automatic classification produced by Chiu et al. [13], which consists of 1149 verbs assigned to 50 classes. Their qualitative evaluation of a small subset of the resource showed that the classes were highly accurate. In this work, we perform a complete manual verification and restructuring of the automatically generated classification, which produces a new, two-level taxonomy of verbs, including 22 top-level classes and 117 subclasses, illustrated in Fig. 1. Next, we carry out two stages of manual annotation, which yield VerbNet-style semantic-syntactic classes, each described by a set of syntactic frames annotated with semantic roles. In the next sections, we first describe the methodology of Chiu et al. [13] and the resultant automatically generated classes, followed by the process of manual verification.

### Automatic verb classification

Chiu et al. [13] proposed an automatic classification method which combines a neural representation learning approach with the classification step. In contrast to previous approaches to automatic induction of verb classes [44–47], the method avoids manual feature engineering, which is time-consuming and requires expert knowledge. Instead, it employs features automatically learned directly from corpora using neural networks, fine-tuned to better capture the semantics of verbs in biomedical texts. Due to the cost-effectiveness of the approach and its demonstrated success in generating high-quality classification output, as validated by human experts in Chiu et al., we chose to leverage its potential in our construction of BioVerbNet.

The classification approach of Chiu et al. involves the following steps. First, they use the method of Vulić et al. [48] to identify the optimal contexts for learning verb representations in the biomedical domain, which creates a context configuration space based on dependency relations between words and subsequently applies an adapted beam search algorithm to find the verb-specific contexts used to generate class-specific word representations. The corpus used in this step includes the PubMed Central Open Access subset [49] and the entire set of PubMed abstracts and consists of approximately 10 billion tokens and 72 million word types. The optimised representations are evaluated on a gold standard biomedical verb similarity dataset, BioSimVerb [6], and are shown to significantly outperform the baseline model (a skip-gram model with negative sampling (SGNS) without class-specific contexts).

Next, the learned representations optimised for biomedical verbs are employed as features for verb classification. To this end, Chiu et al. [13] use a small manually curated VerbNet-style classification of 192 biomedical verbs [50] and expand it with 957 new candidate verbs, assigned to the existing classes using the Nearest Centroid Classifier. The new candidates are derived from BioSimVerb based on their frequency in biomedical journals covering 120 subdomains of biomedicine, which guarantees wide coverage of the resultant classification. The final resource comprises 1149 verbs assigned to 50 classes and additionally provides the most frequent dependency contexts (and example sentences) for each verb.

### Manual verification and extension

Manual verification of the automatic classes from Chiu et al. [13] was carried out in collaboration by two experts, a biologist and a linguist, with postgraduate level of training in their respective fields^1^. They were provided with the original class names and member verbs from the gold standard of Korhonen et al. 2006, the new verbs assigned to the original classes by the classifier, and the set of 10 most frequent dependency contexts for each verb in the sample. For each of the dependency contexts, three example sentences extracted from the corpora and demonstrating the usage of the target verb were also provided.

The goal of the verification process was to examine the automatically generated candidates and check whether they satisfied class membership criteria. Since the aim of this work was to produce a VerbNet-style classification, we adopted an analogous definition of a class as a grouping of verbs with shared semantics and syntactic behaviour, based on the assumption that a verb’s syntactic properties, such as the types of arguments it selects, inform its semantics. This rationale constitutes the foundation of Levin’s [20] (1993) classification of English verbs, which has been extended and refined to create VerbNet.

The verification procedure involved the following steps. First, for each class, the automatically generated new candidate verbs were examined with respect to the original members to ensure semantic coherence; only verbs with meanings similar to the original members were kept. Then, the new candidates were reviewed in terms of their syntactic behaviour, exemplified by the dependency contexts extracted from biomedical corpora, and the class was further refined to include the subset of verbs characterised by common syntactic patterns. Based on the examination of semantic and syntactic properties of the new candidates with regard to the original members, for each new candidate the experts decided if the verb was correctly assigned; otherwise, it was (a) reassigned to another existing class, or (b) a new class was created and the verb in question was assigned to it. During manual verification, some of the original classes were split into smaller subclasses, defined by shared semantics and syntactic behaviour of their members. However, we preserved the information about overlapping semantic properties of groups of subclasses by adopting a two-tier classification structure, with general top-level classes encompassing several narrower subclasses with related semantics. At the end of this process, any verbs which could not be correctly assigned to any of the classes were discarded as noise. Table 1 reports the relevant data statistics, including the number of original classes, the number of reassignments, the number of new classes created in the verification stage, and the number of noisy candidates which were ultimately excluded from the classification.

**Table 1:** Assignment of verbs into classes and sub-classes

	Top-level classes	Sub-classes	Verbs
Automatically assigned verbs	16	48	283 (29.4%)
Manually assigned verbs	-	-	410 (42.7%)
Re-assigned within original sub-classes	-	-	104
Assigned to new sub-classes created within original top-level classes	-	30	93
Assigned to new top-level classes	6	39	213
Total assigned verbs	22	117	693 (72.1%)
Non-assigned verbs	-	-	268 (27.9%)
Total verbs	-	-	961 (100%)

### Comparison with VerbNet

One of the important motivations behind the creation of BioVerbNet is the discrepancy between the general and biomedical domains in terms of language use and words’ distributional properties. This concerns domain-specific words which are frequent in biomedical texts and absent or very rare in the general domain (e.g., *deacetylate, hydroxylate*), as well as words which are common in both domains, but are used in a very narrow, domain-specific sense in the biomedical domain (e.g., *prune, perturb*). In order to examine these discrepancies further, we comparatively analysed the newly created BioVerbNet classes and the existing VerbNet, focusing on the verbs appearing in both.

The results of this analysis support our assumptions: most verbs present in BioVerbNet are missing in VerbNet, and only 39 out of 117 BioVerbNet sub-classes contain one or more VerbNet verbs. In total, 63 of 693 class-assigned BioVerbNet verbs are also present in VerbNet. As expected, sub-classes containing highly biomedical-specific verbs have little or no overlap with VerbNet. For example, sub-class 2.2.1 ‘Biochemical modification’ contains 20 verbs, all of which are specific to BioVerbNet. Other BioVerbNet sub-classes contain a proportion of verbs which are also present in VerbNet, for example, 9.2.2 ‘Cognitive’ contains 39 verbs of which 5 are shared with VerbNet. In all sub-classes, shared verbs form no more than a minority. Moreover, some verbs present in BioVerbNet are characterised by senses that differ from typical usage in the general domain. In Table 2 we provide selected examples.

**Table 2:** Examples of common verbs with senses specific to the biological sciences

	Sense	Example
*silence*	To inactivate expression of a gene.	Eukaryotic cells express small noncoding RNAs to silence target genes
*dampen*	To suppress the immune response.	Propathogenic cells dampen the early T cell response
*scavenge*	To combine with and remove reactive oxygen species.	Antioxidant properties of plants scavenge reactive oxygen species
*prime*	To present antigen to naïve lymphocytes, causing them	These antigens may prime an immune response
	to differentiate.	
*reprogram*	To transform one cell type into a different cell type.	Mash1 and Brn2 reprogram fibroblasts into neurons
*imprint*	To inactivate expression of a gene through methylation.	A period of stimulation could imprint on a T cell a “biochemical memory”
*divide*	To undergo cell division into two or more daughter cells.	Cultures of Tetrahymena pyriformis were induced to divide synchronously
*isolate*	To extract a cell population or substance in a pure form.	We used soft agar to isolate phototrophic bacteria

### Semantic and syntactic annotation

In the original VerbNet classification, each class is accompanied by a set of syntactic descriptions (i.e., syntactic frames), which illustrate the possible surface realisations of a verb’s arguments. For each type of syntactic frame, a sentence example of usage is provided, along with the semantic roles of the verb’s arguments and corresponding semantic predicates (e.g., ‘motion’, ‘has_state’) with temporal and causal subevent structure. In this work, we focus annotation efforts on two main components: semantic roles and syntactic frames. For each BioVerbNet class generated in the manual verification stage, we identify the subset of shared syntactic contexts licensed by all members. Next, we annotate the class-specific syntactic structures with semantic roles and syntactic constituents. We describe the methodology adopted in each step in the following sections.

### Context selection

To identify the subset of shared argument structures and syntactic contexts for each class we utilise the dependency contexts extracted from the PubMed corpus by Chiu et al. [13], which were used in the Manual Verification stage as class membership criteria. Since the dependency contexts of Chiu et al. [13] were automatically generated, they sometimes contained noise and parsing errors. Additionally, some of the contexts were redundant or uninformative with regard to the verb’s argument structure. For example, context types *obj* and *subj#obj* provided redundant information about the verb’s transitive behaviour. Moreover, some dependency contexts included conjunctions (e.g., *and*) or adjunct adverbial and prepositional phrases (e.g., *Physiologic mechanisms regulate hemodynamics during exercise and in heart failure*), which are optional elements and therefore are not considered as characteristic of a given verb’s behaviour. We adopted an iterative context identification protocol, in which for each class, for each class member, we examined the set of 10 most frequent dependency contexts and filtered those redundant or uninformative; then, the remaining contexts were checked against the class members by substituting them one by one into a given context. Only the contexts in which all the class members could participate were kept. After the set of 10 most frequent dependency contexts was reviewed for the first verb belonging to a given class, the procedure was repeated for all the remaining class members.

### Semantic role labeling

The first step of the manual annotation process involved annotating the class-specific syntactic contexts identified in the previous step with semantic roles. Also known as thematic or theta roles, semantic roles describe the underlying relationship between a participant of an event and the main verb in a clause. They capture the differences in verb meaning as reflected in the expression of its arguments and thus provide important generalisations about the interplay of verbs’ semantic and syntactic behaviour, which contribute to the semantic-syntactic mapping.

While consensus has not been reached on the semantic role inventory [51–53], most approaches agree on a number of principal roles and their corresponding definitions, such as *Agent*, the instigator of the action denoted by the predicate, or *Patient*, the entity undergoing the effect of the event [7, 18, 54, 55]. To ensure alignment with VerbNet, we adopted the same set of roles and definitions. However, the discrepancies between the verbs’ common usages in the general and the biomedical domains posed a number of challenges, which required a careful revision of the role assignment criteria, in view of the characteristic properties of biomedical verbs.

#### Challenges and domain-specific roles

The first important difference between the two domains, and consequently the two lexicons, lies in the nature of typical arguments. In VerbNet, the annotated examples predominantly feature canonical role-argument pairings, e.g., animate, intentional Agents, inanimate, concrete objects as Instruments, or human Experiencers. In the biomedical domain, the typical event participants are biological and chemical entities, such as cells, chemical reactions, or hormones. In the sentence *NKT cells mediate autoreactivity*, cells are not only animate, but they also interact with each other and their environment. However, they are not intentional, which is one of the criteria of agency in VerbNet: *Actor in an event who initiates and carries out the event intentionally or consciously, and who exists independently of the event*. Similar considerations involve organs, tumours, or bacteria, which commonly take on the agentive role in biomedical texts. In light of the widespread nature of this phenomenon, we propose a new role, Bio-Agent. We posit it as a subtype of Causer, i.e., *an Actor in an event that initiates and effects the event and that exists independently of the event*, constrained to being a biological process, event or entity as a selectional restriction. A Bio-Agent may deploy a biochemical messenger as an inanimate Instrument, as in the sentence *Endothelial cells release substances that hyperpolarize vascular smooth muscle*, where ‘Endothelial cells’ are Bio-Agents and ‘substances’ are Instruments. In the sentence *Poxviruses deploy genomic accordions to adapt rapidly*, ‘Poxviruses’ are Bio-Agents and ‘genomic accordions’ constitute a biological mechanism functioning as an Instrument. Given the co-presence of canonical Agents (e.g., human actors) in biomedical texts, adopting the role of Bio-Agent helps capture important differences in the characteristic properties of these two types of arguments. Consequently, it allows discrimination between verbs which only permit one type of Agent, thus enabling a more fine-grained classification.

A second difference is that some verbs present in both VerbNet and BioVerbNet are characterised by differences in their typical usage and corresponding semantic roles (Table 3).

**Table 3:** Examples of differences in semantic roles of the arguments of the same verb (underlined) in VerbNet and BioVerbNet

Source	Example sentence	Verb Frame
VerbNet	The couple settled there	Agent V Location
BioVerbNet	Most parasites settle within this area	Patient V {in} Location
VerbNet	The gardener grew that acorn into an oak tree	Agent V Patient {into} Product
BioVerbNet	Alga-free paramecia and symbiotic algae can grow independently	Agent {and} Co-Agent {can} V ADV
VerbNet	He responded to my call	Agent V Theme
BioVerbNet	Plants respond to damage	Agent V Source
VerbNet	The secretary transcribed the speech	Agent V Theme
BioVerbNet	These viruses transcribe their genomes in the nuclei of infected cells	Bio-Agent V Patient {in} Location

In VerbNet, *settle* either describes cognitive agreement and is classified with verbs such as *communicate*, *concur* and *compromise* in the Settle class, or belongs to the Lodge class in the sense ‘to go and live somewhere’ with members such as *dwell, reside* and *stay*. In the sentence *The couple settled there*, ‘the couple’ takes on the role of Agent as the instigator of the action. In biomedical texts, the first (cognitive) sense occurs in analogous contexts, while the second describes the physical movement of objects towards a stationary state. However, these objects are no longer agentive. In BioVerbNet, *settle* is placed in sub-class 19.2.1 ‘Condense’, containing 16 members that include *sediment*, *coalesce* and *agglutinate*. In the sentence *Most parasites settle within this area*, the phrase ‘Most parasites’ takes on the role of Patient since it experiences the effect of the event.

The usage of the verb *transcribe* also differs between VerbNet and BioVerbNet. In VerbNet, *transcribe* describes the copying of speech or text and is placed in a group of 20 members that includes the verbs *chronicle*, *photocopy* and *record*. In biomedical texts, however, *transcribe* is typically used in the sense of DNA transcription, an active process whereby the DNA double helix is unzipped and a complementary strand of mRNA synthesized. In BioVerbNet, *transcribe* is placed in sub-class 17.2.1 ‘Upregulate gene expression’, containing 6 members that include *transactivate*, *upregulate* and *derepress*. The usage of *transcribe* in VerbNet involves a human Agent and the object of the verb is unchanged, therefore a Theme, as in *The secretary transcribed the speech* (Agent V Theme). In BioVerbNet, the agentive role is taken by either a cellular Bio-Agent or a biochemical Force, and the object of the verb is materially altered, therefore a Patient, as in *These viruses transcribe their genomes in the nuclei of infected cells* (Bio-Agent V Patient {in} Location).

### Syntactic frame annotation

The second step of the annotation process consisted in annotating the characteristic frames for each subclass with syntactic constituents. For each frame, we identified word groups functioning as a single unit in the syntactic structure of the sentence (e.g., *NP, VP, AdjP*). These syntactic patterns largely overlap with those used in VerbNet. However, unlike in VerbNet, we have included for certain classes passive constructions and dependent clauses containing the target verb when those syntactic patterns typify the use of those verbs in biomedical text. Table 4 provides examples of syntactic annotation selected from the complete resource.

**Table 4:** Examples of syntactic annotation (verb class members underlined)

Verb sub-class	Example sentence	Syntactic annotation
1.1.2 Suppress	Amine groups quench the excited fluorophore	NP V NP
1.3.0 Increase/Decrease	Hormonal stimuli decline	NP V
	Antibody levels decline rapidly	NP V ADVP
2.2.2 Cleave	The activated caspases truncate procaspase-3	NP V NP
	The conjugated salts chop the cell membrane into pieces	NP V NP PP
2.3.0 Interact	Both drug classes synergize	NP V
	Estrogen may synergize with nonaromatizable androgens	NP V PP
4.1.1 Wash	Subepithelial mucous gland secretions clean the valvular crypts	NP V NP
4.2.0 Precipitate	Specific antisera coprecipitate IGFBP-5	NP V NP
	VITF-A and the viral capping enzyme copurify to near homogeneity	NP V PP
8.1.2 Chemically combine	Nonfunctional receptors could not dimerize	NP V
	Curcumin can chelate metal ions	NP V NP
	Lomefloxacin can chelate with heavy metals	NP V PP
9.5.0 Decipher	We comprehend	NP V
	We decipher the molecular determinants	NP V NP
10.2.0 Score	We classify these diseases as immunodeficiencies	NP V NP PP
	Clinicians classify the patient correctly	NP V NP ADVP
17.2.2 Downregulate gene expression	HDAC4 and MEF2C downmodulate c-jun promoter activity	NP V NP
	MicroRNAs silence the expression of target genes post-transcriptionally	NP V NP PP ADVP
20.1.3 Repair	Hematopoietic stem cells can reconstitute the bone marrow	NP V NP
	Adult zebra fish regenerate their caudal fin following partial amputation	NP V NP PP

## Utility and discussion

### Evaluation

The objective of this evaluation is to apply a standard retrofitting method to change the vector-space of the pretrained word embeddings to better capture the semantics represented by the BioVerbNet classes [56]. We apply retrofitting to our pretrained embeddings (we use the embeddings pre-trained by Chiu et al. [57]). We base our retrofitting approach on the method proposed by Faruqui et al. [58]. Given any pretrained vector-space representation, the main idea of retrofitting is to pull words which are connected in relation to the provided semantic lexicon closer together in the vector space. The main objective function to minimize in the retrofitting model is expressed as

1 $$  \sum\limits_{i=1}^{|V|} \left(\alpha_{i} \left\|{\vec{v}_{i} - \vec{\hat{v}}_{i}}\right\| + \sum_{(i,j)\in S} \beta_{ij} \left\|{\vec{v}_{i} - \vec{v}_{j}}\right\|\right)  $$

where |*V*| represents the size of the vocabulary, $\vec {v}_{i}$ and $\vec {v}_{j}$ correspond to word vectors in a pretrained representation, and $\vec {\hat {v}}_{i}$ represents the output word vector. *S* is the input lexicon represented as a set of linguistic constraints—in our case, they are pairs of word indices, denoting the pairwise relations between member verbs in each BioVerbNet class. For example, a pair (*i*,*j*) in *S* implies that the *i*th and *j*th words in the vocabulary *V* belong to the same verb class.

The values of *α*_*i*_ and *β*_*ij*_ are predefined and control the relative strength of associations between members. We follow the default settings for these values as stated in the authors’ work by setting *α*=1 and *β*=0.05 in all of the experiments. To minimize the objective function for a set of starting vectors $\vec v$ and produce retrofitted vectors $\vec {\hat v}$, we run stochastic gradient descent (SGD) for 20 epochs. An implementation of this algorithm has been published online by the authors;^2^ we used this implementation in this evaluation.

We evaluate our word representations using two established biomedical datasets for text classification: the Hallmarks of Cancer (HOC) [59, 60] and the Chemical Exposure Assessment (CEA) taxonomy [16]. We evaluate each based on their document-level (Pubmed abstract) and sentence-level classifications, where zero or more predefined labels can be assigned for both of these tasks.

The Hallmarks of Cancer depicts a set of interrelated biological factors and behaviours that enable cancer to thrive in the body. Introduced by Weinberg and Hanahan [15], it has been widely used in biomedical NLP, including as part of the BioNLP Shared Task 2013, “Cancer Genetics task” [61]. Baker et al. [59, 60] have released an expert-annotated dataset of cancer hallmark classifications for both sentences and documents in PubMed. The data consists of multi-labelled documents and sentences using a taxonomy of 37 classes.

The Chemical Exposure Assessment taxonomy, introduced by Larsson et al. [16], is an annotated dataset for the classification of text (documents or sentences) concerning chemical risk assessments. The taxonomy of 32 classes is divided into two branches: one relates to assessment of exposure routes (ingestion, inhalation, dermal absorption, *etc*.) and the second to the measurement of exposure bio-markers (biomonitoring). Table 5 details basic statistics for each dataset.

**Table 5:** Summary statistics of the Hallmarks of Cancer (HOC) and the Chemical Exposure Assessment (CEA) datasets

	HOC		CEA	
	Document	Sentence	Document	Sentence
Train	1,303	12,279	2,555	25,307
Dev	183	1,775	384	3,770
Test	366	3,410	722	7,100
*Total*	1,852	17,464	3,661	36,177

We input the retrofitted vectors into a baseline neural network model; we use the convolutional neural network (CNN) model proposed by Kim [62] for text classification tasks. An implementation of this model that was used on both the Hallmarks of Cancer task and the Chemical Exposure Assessment task has been published by Baker et al. [63]; we use this implementation in our experiment. The input to the model is an initial word embedding layer that maps input texts into matrices, which is then followed by convolutions of different filter sizes, 1-max pooling, and finally a fully-connected layer leading to an output Softmax layer predicting labels for text. Model hyperparameters and the training setup are summarized in Table 6.

**Table 6:** Hyper-parameters used in our convolutional neural network

Parameters	Values
Vector dimension	200
Filter sizes	3,4 and 5
Number of filters	300
Dropout probability	0.5
Minibatch size	50
Input size (in tokens)	500 (documents), 100 (sentences)

For both tasks, we use the embeddings^3^ by Chiu et al. [57] without retrofitting as a control baseline, and we evaluate two variations of the BioVerbNet verb classes, the 22 top-level classes, and the 117 subclasses.

Performance is evaluated using the standard precision, recall, and *F*_1_-score metrics of the labels in the model using the one-*vs*-rest setup: we train and evaluate *K* independent binary CNN classifiers (*i.e*. a single classifier per class with the instances of that class as positive samples and all other instances as negatives). Due to their random initialization, we repeat each CNN experiment 20 times and report the mean of the evaluation results to account for variances in neural networks. To address overfitting in the CNN, we use early stopping; testing only the model that achieved the highest results on the development dataset. We apply a two-tailed t-test with *α*=0.05 on the averaged output in comparison with the baseline model.

The results of our valuations are summarised in Table 7 for the HOC task, and Table 8 for the CEA task. We can observe that in both classification tasks, and at both levels of text classification (document and sentence), the retrofitted models outperformed the baseline models with significant results. The more fine-grained 117 subclasses retrofitting improved the document-level classification for both tasks more than the top-level verb classes, whereas for sentence classification the opposite is observed. For the HOC task, Recall benefited substantially from the retrofitting process, whereas for the CEA task both Precision and Recall improved slightly compared to the baseline. The reason behind the difference is likely because the HOC dataset contains classes that are very sparse (with only a small number of examples), and therefore recall would increase more substantially for these classes at the cost of precision; this has also been observed in prior work with the HOC task [56, 63, 64].

**Table 7:** Evaluation results for the Hallmarks of Cancer task (HOC) text classification task

	Document classification			Sentence classification		
Model	Precision	Recall	*F*_1_	Precision	Recall	*F*_1_
Baseline (no retrofitting)	77.8	51.7	62.1	56.8	30.7	39.9
22-classes retrofitted	74.4	62.1	67.7*	49.1	35.8	41.4*
117-subclasses retrofitted	74.8	62.5	68.1*	48.6	35.2	40.8*

The Baseline model is a skip-gram model without any retrofitting. All figures are micro-averages expressed as percentages (Bold denotes the best *F*_1_-score, * denotes statistically significant scores with respect to the baseline)

**Table 8:** Evaluation results for the Chemical Exposure Assessment (CEA) text classification task

	Document classification			Sentence classification		
Model	Precision	Recall	*F*_1_	Precision	Recall	*F*_1_
Baseline (no retrofitting)	89.5	87.1	88.3	66.2	62.8	64.5
22-classes retrofitted	89.9	87.5	88.7*	67.3	62.1	**64.6**
117-subclasses retrofitted	89.2	88.6	88.9*	66.3	60.3	63.2*

Baseline model is a skip-gram model without any retrofitting. All figures are micro-averages expressed as percentages (Bold denotes the best *F*_1_-score, * denotes statistically significant scores with respect to the baseline)

These results demonstrate the utility of BioVerbNet for specialising distributional word embeddings to better capture the properties of verbs in biomedicine and reveal its potential to aid NLP models in tackling domain-specific tasks where accurate verb processing is important.

## Conclusions

This paper introduces BioVerbNet, the first semantic-syntactic classification of biomedical verbs. The resource groups verbs occurring in the PubMed corpus based on shared meaning and syntactic behaviour into 22 top-level and 117 fine-grained classes, each described by a set of characteristic syntactic frames, annotated with semantic roles. To construct BioVerbNet, we started from the output of a neural classification method specialised for biomedical verbs [13], which subsequently underwent manual revision and refinement, as well as semantic-syntactic annotation, by domain and linguistics experts. The resource provides VerbNet-style information for members of each class, including the characteristic syntactic contexts in which they appear and the typical semantic roles taken by their arguments.

BioVerbNet fills the gap in computational lexical resources targeting biomedical verbs currently available and promises to support future work in biomedical NLP. Our evaluation experiments on the task of text classification demonstrated that BioVerbNet can be readily used to support natural language processing models in biomedical tasks. We showed that class membership information from BioVerbNet can be successfully leveraged by retrofitting pretrained word embeddings so that verbs sharing the same BioVerbnet class, and therefore semantic and syntactic behaviour, are pulled closer together in the embedding space. Our retrofitted embeddings outperformed the baseline models by a significant margin on two datasets, Hallmarks of Cancer and Chemical Exposure Assessment taxonomy. Moreover, the resource provides detailed, manually-curated semantic-syntactic annotations for each class, which offer insights into the domain-specific properties of biomedical verbs and can support researchers in developing models capable of nuanced handling of the syntactic and semantic properties of verbs in biomedical texts.

### Future work

BioVerbNet includes 961 biomedical verbs sampled from PubMed corpora, making it the largest lexicon of this kind available in biomedicine. In future work, it can be further extended to cover less frequent verbs and additional classes. Moreover, given that the annotation style in BioVerbNet follows that used in VerbNet, the two resources can be linked at the level of individual verbs appearing in both, thus providing richer information for each entry and enabling easier comparisons of verb behaviour in both domains.

In future work, we will further explore the potential of BioVerbNet to support state-of-the-art NLP systems in solving biomedical tasks. Given the success of BioBERT, we will use our new resource to probe its ability to capture verbal meaning in biomedical texts and compare its performance against our best performing embeddings retrofitted to BioVerbNet class membership information. Moreover, we will investigate the potential of injecting knowledge about biomedical verbs from BioVerbNet into large pretrained encoders to further boost their verbal reasoning capacity in biomedicine. To support future endeavours in biomedical NLP we make our resource freely available to the community at https://github.com/cambridgeltl/bioverbnet.

## Data Availability

The dataset created in this study is available on Github, https://github.com/cambridgeltl/bioverbnet. The datasets used for evaluation are publicly available at: Exposure Assessment taxonomy http://dx.doi.org/10.6084/m9.figshare.4668229 and Hallmarks of Cancer Corpus https://github.com/sb895/Hallmarks-of-Cancer.

## Abbreviations

NLP: Natural Language Processing
UMLS: Unified Medical Language System
BERT: Bidirectional Encoder Representations from Transformers
BOW: Bag-Of-Words
SGNS: Skip-gram model with negative sampling
LSTM: Long short-term memory
ELMo: Embeddings from Language Models

## Footnotes

1 Due to the complementary nature of their expertise, the two experts carried out the verification collaboratively and all decisions were made jointly.

2 https://github.com/mfaruqui/retrofitting

3 https://github.com/cambridgeltl/BioNLP-2016
